# College of American Pathologists Cancer Protocols: From Optimizing Cancer Patient Care to Facilitating Interoperable Reporting and Downstream Data Use

**DOI:** 10.1200/CCI.20.00104

**Published:** 2021-01-13

**Authors:** Vanda F. Torous, Ross W. Simpson, Jyoti P. Balani, Alexander S. Baras, Michael A. Berman, George G. Birdsong, Giovanna A. Giannico, Gladell P. Paner, Jason R. Pettus, Zack Sessions, S. Joseph Sirintrapun, John R. Srigley, Samantha Spencer

**Affiliations:** ^1^ Massachusetts General Hospital, Boston, MA; ^2^ Methodist Hospital, St. Louis Park, MN; ^3^ University of Texas Southwestern Medical Center, Dallas, TX; ^4^ Johns Hopkins University School of Medicine, Baltimore, MD; ^5^ Jefferson Hospital, Allegheny Health Network, Jefferson Hills, PA; ^6^ Emory University School of Medicine at Grady Hospital, Atlanta, GA; ^7^ Vanderbilt University Medical Center, Nashville, TN; ^8^ University of Chicago Medical Center, Chicago, IL; ^9^ Dartmouth-Hitchcock Medical Center, Dartmouth, NH; ^10^ College of American Pathologists, Northfield, IL; ^11^ Memorial Sloan Kettering Cancer Center, New York, NY; ^12^ University of Toronto, Toronto, Ontario, Canada

## Abstract

The College of American Pathologists Cancer Protocols have offered guidance to pathologists for standard cancer pathology reporting for more than 35 years. The adoption of computer readable versions of these protocols by electronic health record and laboratory information system (LIS) vendors has provided a mechanism for pathologists to report within their LIS workflow, in addition to enabling standardized structured data capture and reporting to downstream consumers of these data such as the cancer surveillance community. This paper reviews the history of the Cancer Protocols and electronic Cancer Checklists, outlines the current use of these critically important cancer case reporting tools, and examines future directions, including plans to help improve the integration of the Cancer Protocols into clinical, public health, research, and other workflows.

## INTRODUCTION

As a patient begins a journey of cancer treatment, the pathology report and associated biomarker results represent the starting data set that will drive that patient’s care. While traditionally pathology reports have been narrative-style unstructured text with variable content depending on the institution, the College of American Pathologists (CAP) has provided its Cancer Protocols to guide pathologists in creating synoptic cancer reports. Cancer Protocol–generated reports contain consistent content across institutions, record the data in discrete fields, and allow standardized sharing of these data with other organizations. A computer-readable version of these enables programs to use this information for decision support, prognosis, research, and public health reporting. Rather than manually scanning reports for specific parameters, such as tumor size and grade, computers can query databases for this content and use analytics to provide patient-specific prognostic and therapeutic information. Additionally, the use of data exchange standards allows for interoperability and information sharing under the 21st Century Cures Act.

**Context**

**Key Objective**
To promote understanding of the College of American Pathologists (CAP) Cancer Protocols and their critical use in cancer patient care, and advance collaboration and interoperability in the cancer domain through standardized structured reporting and data exchange.
**Knowledge Generated**
The CAP Cancer Protocols contain guidance and standardized clinical content that support best practices in cancer patient care. The CAP electronic Cancer Checklists (eCCs) allow for that content to be integrated into vendor systems and pathologist workflow to ensure report completeness, and to enable downstream structured data transmission, queries, analytics, quality assurance, research, and use in cancer surveillance and health system planning.
**Relevance**
The use of standardized structured data sets for pathology cancer reporting has been shown to improve patient care and clinical outcomes. The use of the Cancer Protocols and eCCs enhances patient care in addition to interoperability and data exchange through health information technology standards utilization and advancement.

## BACKGROUND

By the mid-1970s, it was recognized that the variation in reporting of cancer specimens by pathologists was problematic. Reporting was often handwritten and narrative, which created the potential to underreport or omit critical data elements needed for patient management. During this time, the American College of Radiology (ACR) had been conducting their own patterns-of-care studies with the goal of improving the quality of care by establishing guidelines for best management practices through peer consensus review and engaged with the CAP around the importance of cancer pathology reports to these efforts.^1^

The CAP Cancer Committee proceeded to form the Patterns of Care Steering Committee in the late 1970s, which later evolved into the Committee on the Pathologist as a Consultant in Cancer Patient Management in the early 1980s. Through the work of these groups, the Cancer Committee conducted their own studies with the aim of standardizing pathologic reporting and establishing the role of the pathologist as a consultant whose objective should be to provide the appropriate information needed for patient treatment.^1^

Work in this area led to the formulation of pathology practice protocols, ultimately culminating in the publication of the first set of Cancer Protocols in 1986 titled “Guidelines for Data to Be Included in Consultation Reports on Breast Cancer, Bladder Cancer, and Hodgkin’s Disease.”^2,3^

Protocols for different cancer types were steadily developed throughout the ensuing years. These included a background documentation section (which was a list of elements to be reported, demographic and clinical information and gross and microscopic findings), explanatory notes, references, and a case summary (checklist).^4-6^

**FIG 1.**
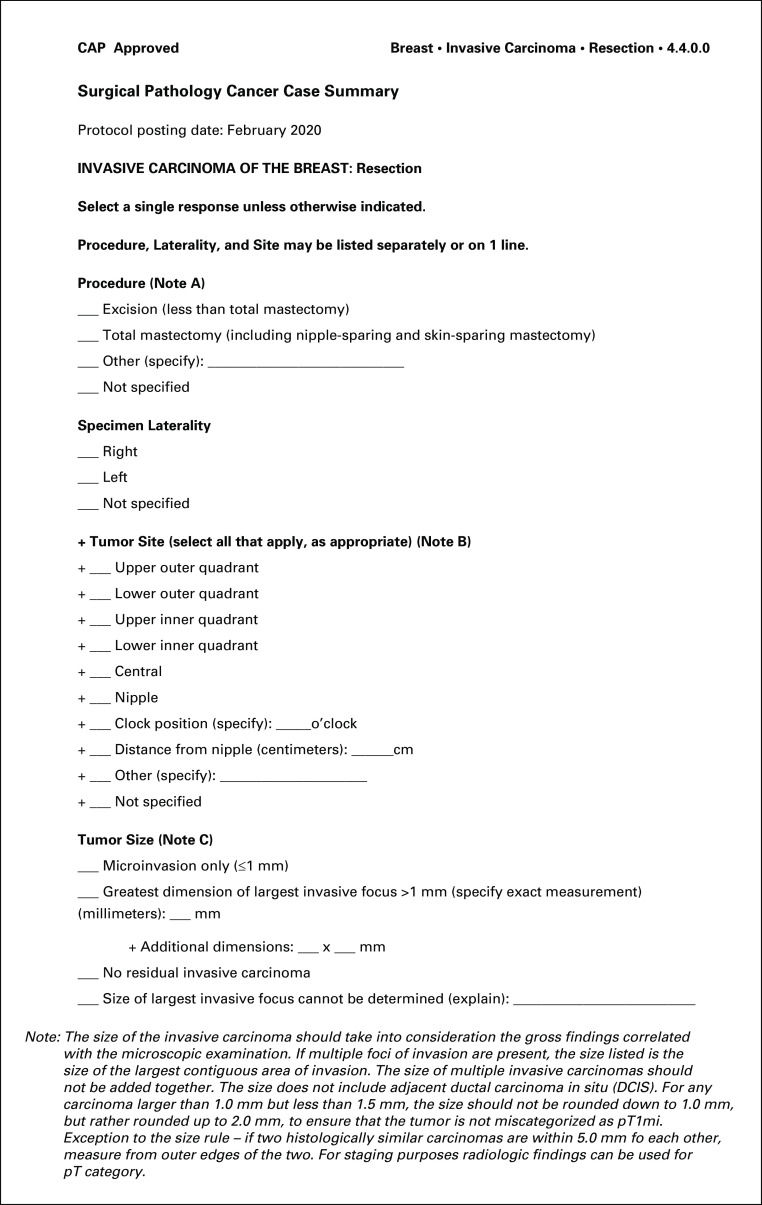
CAP Cancer Protocol for invasive carcinoma of the breast (case summary). The case summary in the CAP Cancer Protocol for Invasive Carcinoma of the Breast^9^ is one of over 100 summaries that are maintained and updated by the CAP on a regular basis as science, medicine, and clinical practice evolve. Each protocol consists of a cover page, case summary, explanatory notes, and references. The case summary contains all elements that a pathologist needs to include in their pathology cancer report in order for it to be considered complete to help optimally direct patient care. The Cancer Protocols are freely accessible via the CAP website^8^. CAP, College of American Pathologists.

Initially, these protocols evoked a mixed response from members of the pathology community, with some citing excessively long reports and others believing that this approach signaled the end of the art of pathology and personalized evaluation.^6^ However, as the complexity of information contained within pathology reports increased over time, it became more accepted that a synoptic style of reporting was needed to ensure accurate and complete documentation of critical information.^7^ While initially the use of the Cancer Protocols was voluntary, the American College of Surgeons Commission on Cancer eventually mandated the reporting of scientifically validated required data elements within the CAP Cancer Protocols for accreditation by 2004, followed by a requirement for CAP laboratory accreditation in 2007.^6^ Since then, the protocols have seen widespread use and now consist of more than 100 case summaries to aid pathologists in their cancer reporting^8^ (Fig 1).

**FIG 2.**
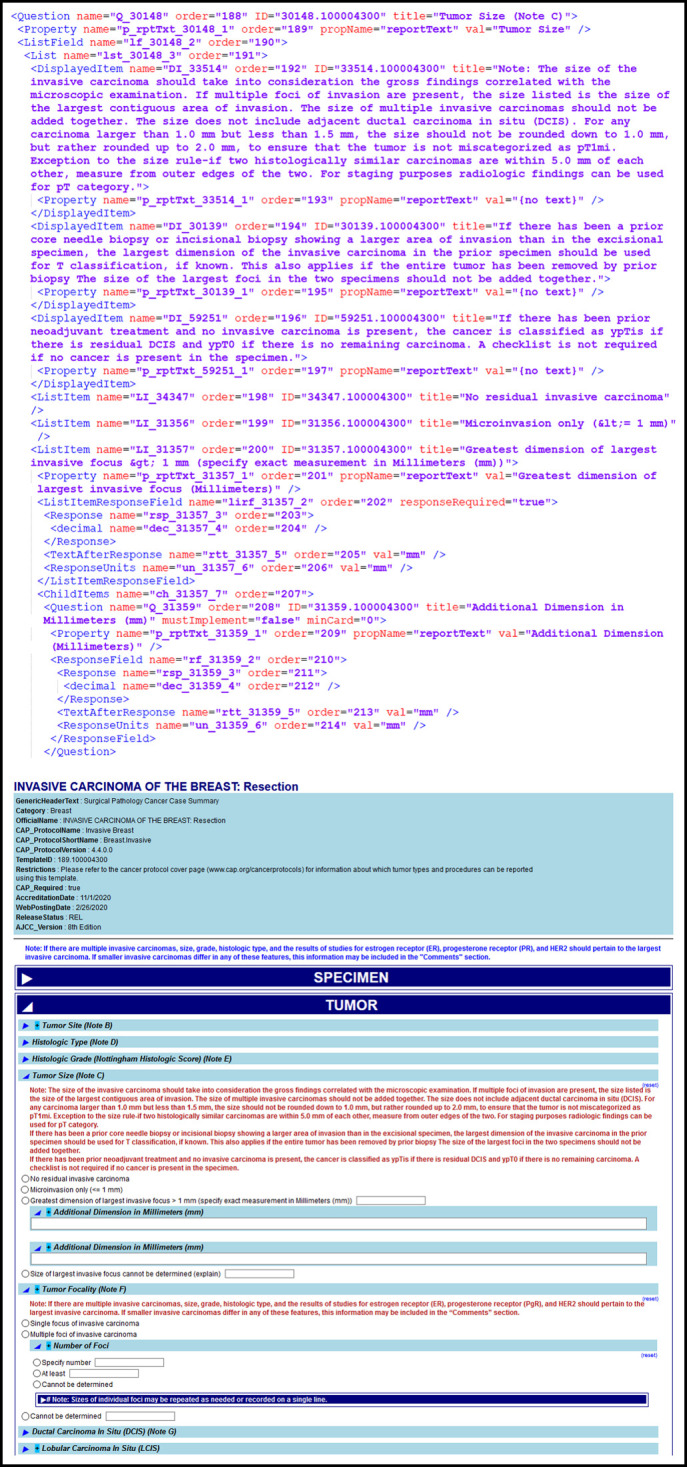
Reporting on invasive carcinoma of the breast cases electronically using the CAP eCC. The case summary in the CAP Cancer Protocol for invasive carcinoma of the breast can be represented in the CAP eCC in computer readable Structured Data Capture XML format. This standard XML technical file is then rendered by vendors and other software systems into a human-readable DEF. CAP, College of American Pathologists; DEF, data entry form; eCC, electronic Cancer Checklists.

The Cancer Protocols were first released in electronic version in 2007 as SNOMED CT (Systematized Nomenclature of Medicine-Clinical Terms, SNOMED International) encoded content^10^ in a common database format. By 2009, the Cancer Protocols were released for the first time in eXtensible Markup Language (XML) format.^6,11,12^ The CAP electronic Cancer Checklists (eCCs) are computer-implementable versions of the Cancer Protocols and Biomarker Templates that can be used for cancer reporting and direct patient care through middleware software, laboratory information systems (LISs), and electronic health records (EHRs). The eCC templates contain question-answer sets and fill-in parameters needed to create the diagnostic patient report. The presence of a question-answer format ensures that the content is explicitly specified and that the needed information is both present and valid. Once filled out in a data entry form (Fig 2), the data can be stored discretely within a vendor or other database. Thus, the eCCs provide several advantages as compared to paper-based synoptic reporting related to the ability to capture and store standardized structured data and ensure completeness of reports.

## CURRENT USE

The Cancer Protocols were first designed as a resource tool for pathologists providing guidelines for cancer reporting and ensuring that all relevant data elements would be reported via standardized terminology. Ultimately, the use of synoptic reports is a quality assurance measure that ensures completeness and consistency: reports contain the necessary diagnostic, prognostic, and predictive elements needed for patient management. Through this standardization, ambiguity is reduced improving communication between pathologists and treating clinicians and allowing for more streamlined patient care.

Initially, the Cancer Protocols were largely based on the work of the Cancer Committee members with multidisciplinary input. Currently, the Protocols are developed and maintained by a multidisciplinary panel of experts (ie, Cancer Protocol Review Panels) with input from members of liaison organizations, the CAP House of Delegates, and other user feedback.

The Pathology Electronic Reporting Task Force (now the Pathology Electronic Reporting [PERT] Committee) was created in 2007^12^ to oversee and guide the development of the electronic version of the Cancer Protocols, including refining the user interface and assisting in data modeling. The work of the PERT Committee further ensures that the cancer reporting information is both standardized and up to date and remains practical and usable for pathologists using electronic reporting through laboratory software systems.

The Cancer Protocols and eCC are under constant revision by the Cancer Protocol authors and review panels, keeping in step with changes in tumor classification systems, staging parameters, and biomarkers. Core elements must be included for the report to be complete and must meet established scientific level of evidence criteria for protocol inclusion, while optional (noncore) elements are proposed parameters that are still being evaluated and may eventually be promoted to a core element. For example, tumor budding in colorectal carcinoma is currently an optional element but could be promoted to a core element for some tumor stages. Definitions and criteria used by pathologists are also continuously updated in the protocols’ Explanatory Notes sections based on feedback from end users and quality studies.

The electronic version of the CAP Cancer Protocols is seeing more widespread use in recent years. The number of licensed full-time equivalent pathologist users of the eCC has grown over the last 6 years from < 1,000 to > 6,400. This represents about 35%-40% of all practicing anatomic pathologists in the United States and Canada. Additionally, approximately 45% of hospitals with > 400 beds in the United States are licensed to use the eCC for diagnostic pathology cancer reporting, and 49 of the 50 states in the United States have laboratories using the eCC.

**FIG 3.**
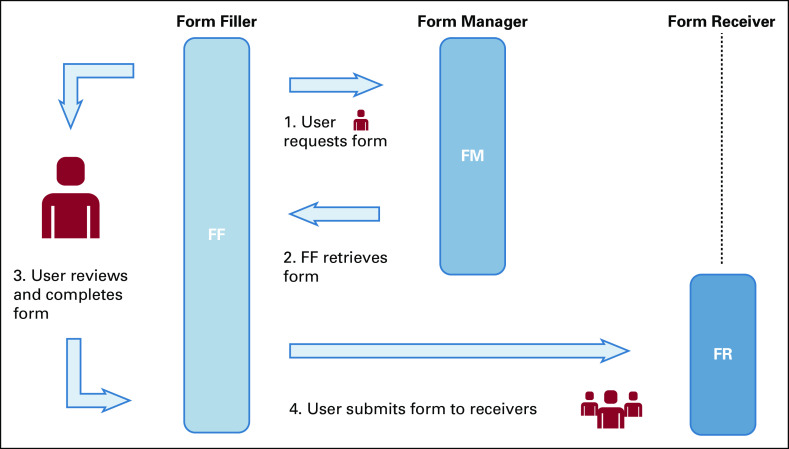
Structured Data Capture (SDC) enables systems to retrieve, display, fill, and submit structured forms to a receiver.^15^ A user, such as a pathologist, requests a specific form to report on an invasive breast cancer case they are reviewing. The correct form is retrieved, and the user reviews and completes the form with all relevant data. The completed form can then be submitted to one or many receivers, who receive these data in the same format in which it was completed. The data can also be transformed into other formats such as a human readable patient report, cancer registry message, or ingested into a database as structured data.^15^

The eCC uses a Structured Data Capture (SDC) format^13^ that offers some notable advantages over paper-based synoptic reporting. The United States Department of Health and Human Services’ Office of the National Coordinator for Health Information Technology created SDC in 2013 to provide a standardized format for clinical data capture, transmission, and sharing.^11^ The CAP eCC was first released in SDC-XML format for vendor implementation and use in February 2019. This format ensures that data are computer-identifiable, retrievable, and processable and uses a standardized data set lexicon^14^ (Fig 3).

Reporting using structured data is advantageous as it facilitates easier case retrieval and data transfer by supporting extraction of data elements with standard and discrete values. Without this, there is a lack of uniformity in cancer reporting that can affect the ability to extract data because of the diversity of terms used or nonuniform selection of reportable elements. Although natural language processing (NLP) tools and machine learning capabilities^16^ have become more sophisticated, they are still not sufficiently powerful to successfully and accurately mine all unstructured cancer data with consistent precision and sensitivity.^17,18^ The use of the CAP Cancer Protocols can improve the yield of NLP by standardizing the language^19^; however, this does not entirely solve back-end data harmonization issues. Ideally, information is captured as discrete data upfront with a controlled vocabulary, such as with the Cancer Protocols and eCC, preemptively eliminating misinterpretation of the data that may occur during manual abstraction, NLP, and coding translation processes. Fidelity of these report data is critical for patient management and for numerous downstream uses including cancer surveillance, research, education, analytics, quality assurance, and health system planning.

Dependence on clinical vendor implementation adds another variable to standardization, and so, greater attention to vendor engagement is now helping to mitigate this. Over the past 2 years, most pathology and LIS vendors have successfully implemented the eCC in SDC format. Collaborative efforts with vendors are ongoing to render the Cancer Protocols efficiently and accurately as data entry forms and reporting tools, with a primary goal to reduce the cycle time from protocol release to implementation. One of the challenges is that many vendors are required to transform the current SDC format into their proprietary system. Improvement of that process and adoption of newer technologies to address any shortcomings is an ongoing goal.

Additionally, significant efforts are ongoing through a cooperative agreement with the Centers for Disease Control and Prevention (CDC) National Program of Cancer Registries (NPCR) and a recent grant with the California Department of Public Health (CDPH) California Cancer Registry. The collective work toward the development and implementation of standardized structured reporting (SSR) across laboratories and health systems shares a goal of automated, direct transmission of cancer case data to centralized cancer registries.^20^ Activities include reconciling clinical cancer reporting with cancer registry data collection standards, cooperative work on metric development and measurement for cancer reporting from laboratories to registries, promotion of use of technical standards such as SDC for more automated transmission of cancer report data to registries, and alignment on biomarker and other key data capture components.

The CAP is also engaging with the Ontario Health—Cancer Care Ontario, the North American Association of Central Cancer Registries (NAACCR), the National Cancer Institute—SEER, the American Joint Committee on Cancer (AJCC), ASCO, International Collaboration on Cancer reporting, the University of Nebraska Medical Center, the ACR, and the WHO International Agency for Research on Cancer to improve alignment of release scheduling, to develop common coding practices (eg, International Classification of Diseases for Oncology, Third Edition (ICD-O-3), Minimal Common Oncology Data Elements, and SNOMED CT), and to adjust the flow and automation of cancer case identification both for clinical use and for capture into cancer surveillance systems.

## FUTURE

Continuing to build on the current momentum to improve the data capture, reporting, and portability, there are plans to further use the expanded capabilities of the SDC format for both direct patient care and downstream data use purposes. One of the goals is to provide rule capability within the SDC format that will allow dependencies of different questions and answers to be evaluated in real time when the form is being filled out. Stage classification, for instance, can be calculated from answers to the specific staging parameters that are collected while filling out the data entry form that creates the report. Setting relationships between SDC question and answer metadata enables the program to automatically calculate stage classification and check for inconsistencies within the completed cancer patient pathology report. This functionality is similar to that of consumer tax preparation software, where branching logic changes questions that are relevant to the respondent according to their responses and may constrain possible answers to be consistent with the other reported elements. The challenge is that the rule information is supplied generically within the SDC format, but successful implementation relies on the specific vendor system capabilities. Vendors could choose to offload some of these tasks to an outside application program interface or other similar technologies. This would streamline data entry by the pathologist and ensure internally consistent data in the pathology report.

As noted in the background review, the initial electronic protocol version used SNOMED CT as the basis for terminology. That tight coupling was abandoned because of the need for new terms that were not available in SNOMED CT. However, there is an ongoing effort to map CAP cancer protocol data elements to other terminologies, including ICD-O-3 and NAACCR Site Specific Data Items, and a project to SNOMED-encode the CAP Cancer Protocols, including creation of new SNOMED CT precoordinated observable concepts for accurate mapping.^21^

Importantly, there is a significant push to address the future of downstream interfaces and systems to make the best use of these data. The most obvious model for this is public health for cancer reporting and quality measures that is currently in place in Canadian provinces, such as Ontario, in parts of California, and in work with the CDC NPCR. Quality indicators generated from structured pathology databases can be used to compare practice patterns across jurisdictions, institutions, and even individual providers.^22-24^

However, future use of these electronic data applies to both patient care with real-time implementation for treatment and research use for analysis of discrete pathology report data. For example, Sluijter et al^25^ looked at whether using SSR could affect clinical outcomes in patients with colorectal cancer. They determined that SSR improved patient care by providing more complete, higher quality reports that led to more effective care delivery and better patient outcomes. In addition, there is work underway at the CAP to develop frameworks for cancer biomarker reporting that would span tumor types for both immunohistochemistry (eg, programmed death-ligand 1) and genomic measures (eg, Tumor mutational burden - High and Microsatellite instability - High) and as to consider how to most effectively combine molecular results and digital images to optimize clinical review of these data to help inform treatment options. Discrete data for these elements can also allow for computational analysis to assist the treating physician with the selection of appropriate therapies and provide prognostic data useful in patient care.^26^

The current interfaces for public health follow the NAACCR Volume V standard in Health Level Seven International (HL7) v2.x messages for transmitting discrete pathology report data.^27^ Future possibilities include interfaces based on the Integrating the Healthcare Enterprise (IHE) SDC profile,^28^ including HL7 Fast Healthcare Interoperability Resources FHIR-based transmissions (eg, SDC on FHIR).^14,29^ These evolving technical profiles have been tested in IHE-Connectathon activities and demonstrated at Healthcare Information and Management Systems Society, Inc. Interoperability Showcases to validate their robust functionality and translation into practice in the real world. The desired application and outcome would be reconciling EHR, SDC, and other future data format use with cancer registry software and other surveillance tools, allowing for the automated push, transfer, and ingestion of these data in an accepted standardized format. Even as this area is undergoing sustained growth and development, current adoption of newer technologies has been slow because of the persistence of legacy systems with outdated interfaces which still meet institutional shorter term clinical needs.

Additionally, genetic analysis has become critical to defining some tumors and to identify specific therapies. For some cancers, there is a standard set of testing, and synoptic reports have been created to allow these data to be recorded as discrete data. This field is rapidly expanding such that having these data as discrete data will be necessary to allow decision support to analyze the burgeoning amount of data and to assist the clinician in identifying possible options. The SDC format is capable of handling a variety of data structures that may be needed to collect these types of data, but determining an agile standard to collect and transport these data is still a work in progress.

**FIG 4.**
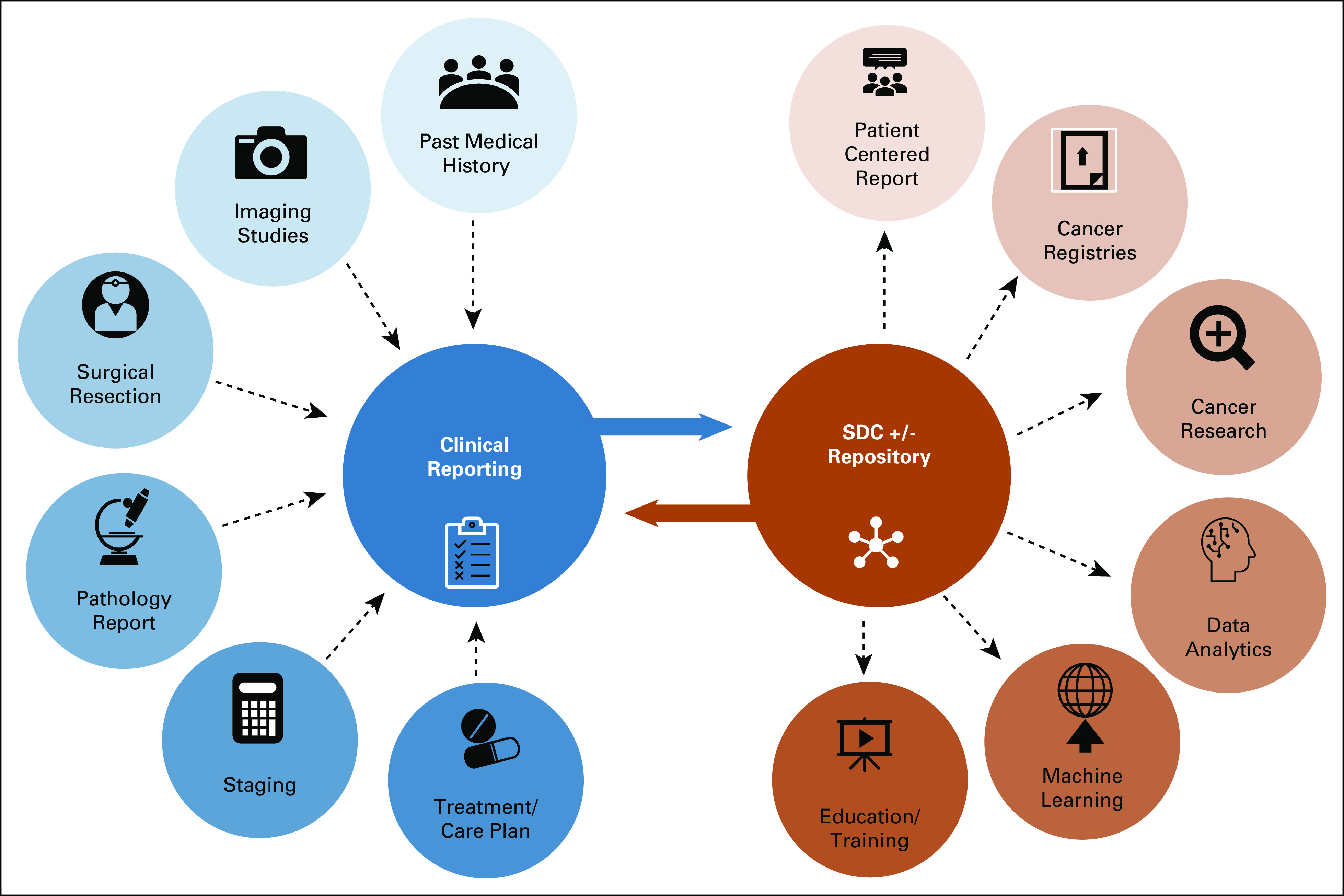
Vision of an interoperable future for cancer data exchange, patient care, and downstream data use. Multidisciplinary reports are currently issued in varied formats, and translation and integration of these may be limited by the allowable outputs from their electronic health record systems. We envision harmonizing reporting structures using a technical standard such as Structured Data Capture (SDC) to be used in direct patient reporting and to communicate these data accurately and effectively to downstream data users.

A vision for the future of cancer data exchange shares in the basic principles behind the eCC, as evolution toward an automated system of cancer reporting aiding pathologists and clinicians in their workflow and in patient care that is also synchronized with downstream data use needs. The SDC framework can help achieve this through integration into vendor systems to capture pretreatment clinical information and export data to downstream systems (Fig 3). To have a successful data exchange ecosystem where clinical cancer reports are automatically subsumed by the cancer surveillance and research communities, we must continue to strive for an agreed-upon reporting structure based on standardized content and technical frameworks (Fig 4). These technical frameworks may also include nonrelational database structures, such as an XML database, that could retain the XML object and use newer technologies for querying.

## SUMMARY

The CAP Cancer Protocols and eCCs have evolved from paper forms to a relational database to the current SDC-XML format which allows metadata to manage the complex relationships between multiple tumor data elements. Cancer Protocol’s electronic reporting and adoption has steadily grown in the clinical domain, with ongoing evolution of use by downstream data consumers such as the cancer surveillance community. This trend is continuing to close the gap, providing accurate and detailed cancer reports in a computable form needed for the next generation of data-driven cancer care and personalized medicine.
